# Supplementary material for: Comparative multiomics analysis of cell physiological state after culture in a basket bioreactor
Source: Sci Rep. 2022 Nov 23;12:20161. doi: 10.1038/s41598-022-24687-4 (PMC9686226; doi:10.1038/s41598-022-24687-4)
Supplement: Supplementary file 1 — Supplementary Information 1. [file 41598_2022_24687_MOESM1_ESM.zip › raw data/Proteomic raw data/6.KEGGenrichment/EG.vs.CG/ALL/pathway/src/04722.html]

map04722


- K02158

- Up regulated proteins

A0A0D9R491(Description:Uncharacterized protein;Gene:BAD;log2FC:inf)

- K12388

- Down regulated proteins

A0A0D9S6J4(Description:Sortilin 1;Gene:SORT1;log2FC:-1.1283)

- K12464

- Down regulated proteins

A0A0D9RJT1(Description:MAGE domain-containing protein;Gene:MAGED1;log2FC:-1.2290)

Close
